# Supplementary material for: Factors associated with admission to the intensive care unit and mortality in patients with COVID-19, Colombia
Source: PLoS One. 2021 Nov 19;16(11):e0260169. doi: 10.1371/journal.pone.0260169 (PMC8604321; doi:10.1371/journal.pone.0260169)
Supplement: S1 Table — (DOCX) [file pone.0260169.s001.docx]

**S1 Table**. Comparison of some sociodemographic and clinical variables among the cities of care of a group of patients infected by SARS-CoV-2, Colombia.

| **Characteristics** | **Bogotá** | | **Cali** | | **Pereira** | | **Popayan** | | **p*** |
| --- | --- | --- | --- | --- | --- | --- | --- | --- | --- |
|  | **n=306** | **%** | **n=302** | **%** | **n=100** | **%** | **n=72** | **%** |  |
| Male sex | 171 | 55.9 | 202 | 66.9 | 63 | 65.0 | 41 | 56.9 | 0.009 |
| Age, median (IQR) | 55.5 (44.0 - 66.3) | | 60.0 (49.0 - 71.0) | | 54.0 (43.0 - 66.8) | | 53.5 (37.0 - 68.5) | | <0.001 |
| ≥65 years | 90 | 29.4 | 122 | 40.4 | 29 | 29.0 | 21 | 29.2 | 0.001 |
| Charlson index, median (IQR) | 1 ( 0 - 3) | | 2 (1 - 3) | | 2 (0 - 4) | | 2 (0 - 3) | | 0.101 |
| ≥3 points | 91 | 29.7 | 110 | 36.4 | 38 | 38.4 | 23 | 32.4 | 0.196 |
| NEWS2 score, median (IQR) | 6 (4 - 7) | | 7 (6 - 9) | | 6 (5 - 8) | | 5 (1.3 - 8) | | <0.001 |
| ≥7 points | 117 | 38.2 | 172 | 57.0 | 48 | 48.0 | 26 | 36.1 | <0.001 |
| Severe pneumonia | 149 | 48.7 | 193 | 63.9 | 58 | 58.0 | 28 | 38.9 | <0.001 |
| Acute respiratory distress syndrome | 160 | 52.3 | 264 | 87.4 | 65 | 65.0 | 20 | 27.8 | <0.001 |
| Admission to ICU | 63 | 20.6 | 144 | 47.7 | 39 | 39.0 | 7 | 9.7 | <0.001 |
| Invasive mechanical ventilation | 52 | 17.0 | 116 | 38.4 | 30 | 30.0 | 5 | 6.9 | <0.001 |
| Deceased | 59 | 19.3 | 112 | 37.1 | 24 | 24.0 | 8 | 11.1 | <0.001 |

IQR: Interquartile range; * comparison between Cali and the other cities
